# Alterations of STEP46 and STEP61 Expression in the Rat Retina with Age and AMD-Like Retinopathy Development

**DOI:** 10.3390/ijms21155182

**Published:** 2020-07-22

**Authors:** Darya V. Telegina, Elizabeth A. Kulikova, Oyuna S. Kozhevnikova, Alexander V. Kulikov, Tatyana M. Khomenko, Konstantin P. Volcho, Nariman F. Salakhutdinov, Nataliya G. Kolosova

**Affiliations:** 1 Institute of Cytology and Genetics, Siberian Branch of Russian Academy of Sciences (SB RAS), Pr. Lavrentyeva 10, 630090 Novosibirsk, Russia; kulikova@bionet.nsc.ru (E.A.K.); oidopova@bionet.nsc.ru (O.S.K.); v_kulikov@bionet.nsc.ru (A.V.K.); kolosova@bionet.nsc.ru (N.G.K.); 2 N.N. Vorozhtsov Institute of Organic Chemistry, SB RAS, 9 Lavrentieva Avenue, 630090 Novosibirsk, Russia; chomenko@nioch.nsc.ru (T.M.K.); volcho@nioch.nsc.ru (K.P.V.); anvar@nioch.nsc.ru (N.F.S.)

**Keywords:** age-related macular degeneration, striatal-enriched protein tyrosine phosphatase, *Ptpn5*, STEP46, STEP61, TC-2153, OXYS rats

## Abstract

Tyrosine phosphatase STEP (striatal-enriched tyrosine protein phosphatase) is a brain-specific protein phosphatase and is involved in the pathogenesis of many neurodegenerative diseases. Here, we examined the impact of STEP on the development of age-related macular degeneration (AMD)-like pathology in senescence-accelerated OXYS rats. Using OXYS and Wistar rats (control), we for the first time demonstrated age-dependent changes in *Ptpn5* mRNA expression, STEP46 and STEP61 protein levels, and their phosphatase activity in the retina. The increases in STEP protein levels and the decrease of total and STEP phosphatase activities in the retina (as compared with Wistar rats) preceded the manifestation of clinical signs of AMD in OXYS rats (age 20 days). There were no differences in these retinal parameters between 13-month-old Wistar rats and OXYS rats with pronounced signs of AMD. Inhibition of STEP with TC-2153 during progressive AMD-like retinopathy (from 9 to 13 months of age) reduced the thickness of the retinal inner nuclear layer, as evidenced by a decreased amount of parvalbumin-positive amacrine neurons. Prolonged treatment with TC-2153 had no effect on *Ptpn5* mRNA expression, STEP46 and STEP61 protein levels, and their phosphatase activity in the OXYS retina. Thus, TC-2153 may negatively affect the retina through mechanisms unrelated to STEP.

## 1. Introduction

Striatal-enriched tyrosine protein phosphatase (STEP), encoded by the *Ptpn5* gene, is a neuron-specific phosphatase that regulates synaptic function and plasticity, whereas its dysregulation is associated with neurodegenerative diseases including Alzheimer’s disease (AD) [1,2]. High levels of STEP are present in human postmortem brain samples and animal models of Alzheimer’s disease [3]. For interaction with substrates, STEP isoforms should contain a kinase-interacting motif (KIM). Only STEP46, a cytosolic isoform, and STEP61, a membrane-associated isoform, contain the KIM domain. It has been shown that STEP isoforms are expressed in such brain regions as the cortex, striatum, amygdala, hippocampus, and optic nerve [3]. The retina is a part of the brain and forms from the embryonic forebrain during early development of the nervous system. The embryonic forebrain gives rise to many structures in the diencephalon (including the thalamus and hypothalamus) and in the cerebral cortex with underlying white matter and basal ganglia. It has been reported that five STEP isoforms (STEP28, STEP30, STEP33, STEP46, and STEP64–66) are expressed in the normal adult rat retina [4]. Nonetheless, there are no data on age-related alterations of STEP expression in the retina and on its role during the development of neurodegenerative retinal diseases associated with aging such as age-related macular degeneration (AMD).

AMD is a late-onset neurodegenerative retinal disease that has become a major cause of blindness in developed countries. AMD shares several clinical and histopathological features with AD and some researchers regard AMD as AD of the eye [5]. The two diseases share similar environmental risk factors: cigarette smoking, hypertension, hypercholesterolemia, atherosclerosis, obesity, and an unhealthy diet. Cellular pathology of AMD and AD is associated with increased oxidative stress, inflammation, and impaired proteasomal and lysosomal function, which evoke the formation of intra- and extracellular deposits. The detrimental deposits consist of aggregated proteins largely similar between the two diseases [5]. AMD is classified into dry (atrophic) AMD and wet AMD depending on the presence of choroidal neovascularization. Most AMD cases start as the dry type, and in 10–20% of patients, it progresses to the wet type. Although the introduction of antiangiogenesis therapy has helped to prevent blindness and restore vision in wet AMD, there is still no effective treatment of dry AMD (~90% of all cases) [6]. The decline of the choroid, retinal pigment epithelium (RPE), and Bruch’s membrane, all of which are typical for aging, underlies AMD pathogenesis; however, the mechanism of their transition to pathological processes remains unclear [7]. Moreover, alterations of additional cell types, such as ganglion, amacrine, bipolar, and glial cells, contribute to the pathogenesis of AMD.

Our study is aimed at determining a possible contribution of age-related STEP46 and STEP61 expression and activity alterations to the development of AMD using senescence-accelerated OXYS rats that spontaneously develop a phenotype similar to human age-related disorders including AD-like pathology and AMD-like retinopathy. In the last decade, convincing arguments have been published confirming that OXYS rats (created at the Institute of Cytology and Genetics, Novosibirsk, as described earlier [8]) develop retinopathy with clinical, morphological, and molecular features similar to those of human AMD. The retinopathy that develops in OXYS rats even at a young age corresponds to the dry atrophic form of AMD in humans. Furthermore, neovascularization develops in some (~10–20%) of these rats with age. Already by the age of ~3–4 months, 100% of OXYS rats develop clinical signs of retinopathy against the background of a reduction in the transverse area of the retinal pigment epithelium (RPE), impairment of choroidal microcirculation, and retinal thinning [8]. The progression of these abnormalities in OXYS rats with age is accompanied by a significant reduction in the thickness of the photoreceptor cell layer and a decrease in the number of photoreceptor cell nuclei in the outer nuclear layer (ONL), especially in the central part of the retina [9,10]. The significant pathological changes in the RPE manifest themselves as excessive accumulation of lipofuscin and amyloid in the RPE regions and disturbances in the morphology of the RPE sheet, including an increase in the proportion of multinucleated cells, hypertrophy, distortion of cell shape, and reactive gliosis [11].

In this study, we analyzed age-related changes of STEP46 and STEP61 expression and activity in the retina of OXYS rats at the age of 20 days (the preclinical assay of AMD-like retinopathy) and at age 13 months (assessment of the progressive stage of disease) using age-matched Wistar rats as a control. In addition to elucidating the role of STEPs in the development of AMD-like pathology in the retina, we estimated the effects of prolonged STEP inhibition in senescence-accelerated OXYS rats. As a tool, we used the well-known STEP inhibitor TC-2153 (8-[trifluoromethyl]-1,2,3,4,5-benzopentathiepin-6-amine hydrochloride) [12,13], which was discovered by us as the first orally available synthetic pentathiepin [14]. Recently, it was shown that relatively brief treatment with TC-2153 is effective in reversing cognitive and memory deficits in a mouse model of AD. The anti-STEP drug TC-2153 has been demonstrated to improve the cognition and memory in a mouse model of AD [3] and to reverse motor and cognitive deficits in phencyclidine-treated mice [15]. On the other hand, the administration of TC-2153 in these studies was brief (from one to two weeks). Here, we studied the effects of prolonged (4-month) treatment with TC-2153 started at the age of nine months on the retina of OXYS rats.

## 2. Results

### 2.1. Age-Dependent Alterations of Ptpn5 mRNA Expression in the Retina of OXYS and Wistar Rats

**Figure 1:**
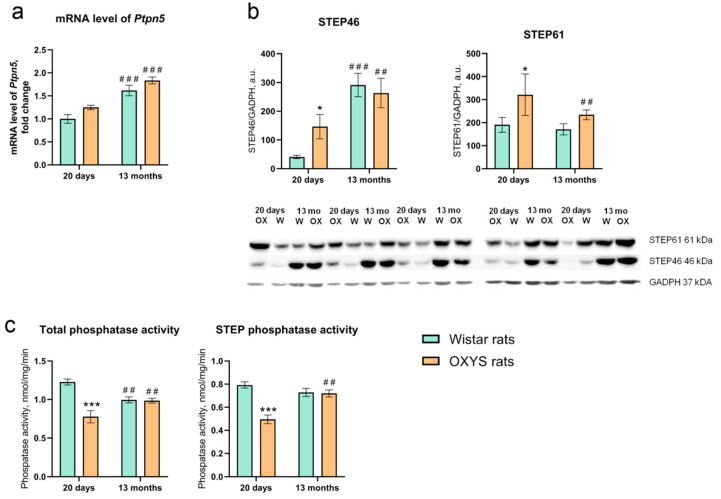
Age-dependent alterations of *Ptpn5* mRNA expression (gene expression is presented as a fold change relative to 20-day-old Wistar rats) (**a**), STEP46 and STEP61 protein levels (**b**), and total phosphatase and STEP phosphatase activities (**c**) in the retina of OXYS rats compared with Wistar rats at 20 days and 13 months of age. Data are presented as the mean ± SE, *n* = 5. Significant differences between Wistar and OXYS rats: * *p* < 0.05; 1; *** *p* < 0.001; 20 days vs. 13 months for the same strain; ^##^ *p* < 0.01; ^###^ *p* < 0.001.

We used primers specific for the 15th and 16th exons, encoding parts of isoforms STEP46 and STEP61, to evaluate the *Ptpn5* mRNA level in the retina. Nevertheless, we noted a significant effect of age (F_1,24_ = 22.10, *p* < 0.001) but not genotype (i.e., strain; F_1,24_ = 2.28, *p* > 0.05) or age × genotype interaction (F_1,24_ = 0.41) on the *Ptpn5* mRNA level in the retina. The 13-month-old rats had an elevated level of *Ptpn5* expression in comparison with 20-day-old animals (Figure 1a).

### 2.2. Alterations of STEP46 and STEP61 Protein Levels with Age and the Development of AMD-Like Retinopathy

We next investigated the levels of STEP46 and STEP61 isoforms in the retina by Western blot analysis. Two-way analysis of variance (ANOVA) showed that there was a significant effect of age (F_1,15_ = 21.92, *p* < 0.001) but not genotype (F_1,15_ = 0.97) on the STEP46 level in the retina. Factors age and genotype interacted (F_1,15_ = 2.90, *p* < 0.05), owing to the finding that at the age of 20 days, the STEP46 level was more than 3-fold higher in OXYS rats than in Wistar rats. From 20 days of age to 13 months, the STEP46 level increased ~7-fold in Wistar rats, while there was a ~2-fold increase in this parameter in OXYS rats.

There was a significant effect of the genotype (F_1,15_ = 9.11, *p* < 0.01) and a slight effect of age (F_1,15_ = 3.98, *p* = 0.064) but no effect of an age × genotype interaction (F_1,15_ = 2.35, *p* > 0.05) on the STEP61 protein level in the retina. The STEP61 level was ~1.5-fold higher in OXYS rats than in Wistar rats (*p* < 0.05) at the age of 20 days, and there was a tendency for this parameter to be higher in OXYS rats at the age of 13 months (*p* = 0.07). By age 13 months, the STEP61 level in the retina of OXYS rats decreased significantly (*p* < 0.05).

### 2.3. Total Phosphatase and STEP Phosphatase Activities in the Retina of OXYS and Wistar Rats

According to two-way ANOVA, both total and STEP phosphatase activities (Figure 1c) were dependent on the genotype (F_1,24_ = 26.05, *p* < 0.001, and F_1,24_ = 20.93, *p* < 0.001, respectively). Moreover, an age × genotype interaction effect was observed for both activities (total phosphatase: F_1,24_ = 23.61, *p* < 0.001; STEP: F_1,24_ = 18.56, *p* < 0.001). We detected an influence of age on STEP activity (F_1,24_ = 5.76, *p* < 0.05) but no effect of this factor on the total phosphatase activity (F_1,24_ = 0.06). Of note, we found that at the age of 20 days, both total and STEP phosphatase activities were significantly lower in OXYS rats than in Wistar rats (*p* < 0.001 for both). At the age of 13 months, the phosphatase and STEP activities of OXYS rats increased, while in Wistar rats, their activities decreased in comparison to those of 20-day-old rats. By age 13 months, the difference between the genotypes disappeared (Figure 1c).

### 2.4. Effects of TC-2153 on the Ptpn5 mRNA level, STEP46 and STEP61 Protein Levels, and Total Phosphatase and STEP Activities in the OXYS Rat Retina

STEP inhibitor (TC-2153) exposure was started at the age of nine months when the clinical manifestations of retinopathy in OXYS rats corresponded mostly to the 1st stage of retinopathy. Four-month intake of TC-2153 produced no effects on Ptpn5 mRNA and protein levels and total phosphatase and STEP activities (Table 1, Figure S1).

### 2.5. Effects of the STEP Inhibitor TC-2153 on Retinal Apoptosis and Thickness in OXYS Rats

**Figure 2:**
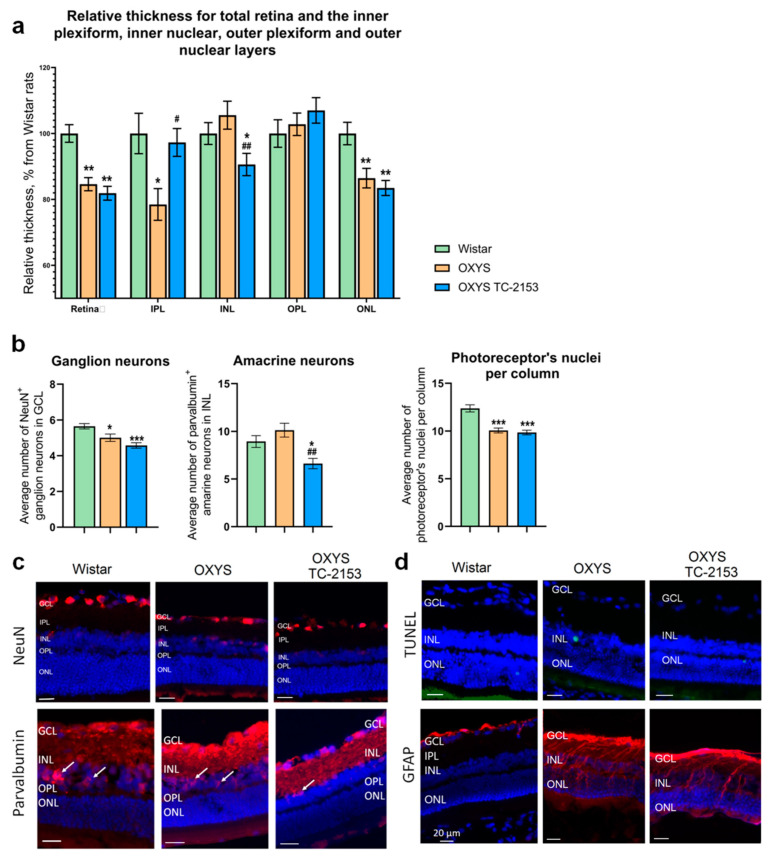
Effects of the STEP inhibitor (TC-2153) on retinal thickness metrics (**a**) and on the amounts of ganglion neurons, amacrine neurons, and photoreceptors (**b**) in 13-month-old OXYS rats. Data are presented as the mean ± SE, *n* = 4. Significant differences as compared to Wistar rats: * *p* < 0.05, ** *p* < 0.01, *** *p* < 0.001; OXYS rats as compared to TC-2153–treated OXYS rats: ^#^ *p* < 0.05, ^##^ *p* < 0.01. (**c**) Representative images of retinas stained with antibodies against NeuN, parvalbumin, and GFAP and (**d**) apoptotic-cell detection by the TUNEL assay in Wistar and OXYS rats. Scale bar = 20 µm. Cell nuclei were stained with 4′,6-diamidino-2-phenylindole (DAPI). Arrows indicate amacrine neurons. GCL, ganglion cell layer; INL, inner nuclear layer; IPL, inner plexiform layer; and ONL, outer nuclear layer.

Despite the absence of an influence of TC-2153 on STEP expression and phosphatase activity, certain structural and functional changes in the retina of OXYS rats as a result of this treatment were identified. First, we examined the thickness of the retina and retinal layers. In agreement with our previous study [8], our data indicated that the relative thickness of the retina in the ganglion cell layer (GCL), outer plexiform layer (OPL), ONL, inner plexiform layer (IPL), and inner nuclear layer (INL) was less in OXYS rats than in Wistar rats. We detected decreasing relative thickness of the retina, IPL, and ONL in OXYS rats as compared to Wistar rats. TC-2153 did not affect relative thickness of the retina and ONL in OXYS rats. At the same time, in the TC-2153–treated OXYS rats, the relative thickness of the IPL was 20% greater (*p* < 0.01), and the relative thickness of the INL was lower than that in untreated OXYS rats (*p* < 0.01; Figure 2a). Next, we assessed apoptosis of retinal cells by the terminal deoxynucleotidyl transferase dUTP nick end labeling (TUNEL) assay. Administration of TC-2153 did not seem to cause any major quantitative change in the apoptosis process in the OXYS retina. In agreement with our previous study, we detected a few isolated TUNEL-positive cells in Wistar and OXYS rats [16].

### 2.6. Effects of the STEP Inhibitor TC-2153 on the Amounts of Amacrine Neurons, Ganglion Neurons, and Photoreceptors

Antibodies against parvalbumin stain mostly AII amacrine cells, along with a few wide-field amacrine cells, a population of bipolar cells in the INL, and a few cells in the GCL [17]. We quantified parvalbumin-positive cells in the INL. Our data revealed no difference between Wistar and OXYS rats, but there was a significant decrease in the number of parvalbumin-positive cells in TC-2153–treated OXYS rats as compared to both Wistar and OXYS control rats (*p* < 0.01; Figure 2b,c).

For the detection of mature ganglion cells, we employed an antibody against NeuN (neuronal nuclear antigen). Quantitative analysis showed that in OXYS rats, the average number of NeuN-positive cells in the GCL was lower than that in Wistar rats (*p* < 0.05), whereas the treatment with TC-2153 had no influence on this parameter. In the ONL, nuclei are typically arranged in columns in well-oriented sections. We determined the average number of nuclei per column in the same regions where the thickness measurements were performed. We detected a significantly lower number in untreated OXYS rats than in Wistar rats (*p* < 0.001) as well as an effect of TC-2153 on the number of ONL nuclei per column in OXYS rats (*p* < 0.001; Figure 2b,c).

### 2.7. Effects of the STEP Inhibitor TC-2153 on Macroglia and NGF and BDNF Expression

**Figure 3:**
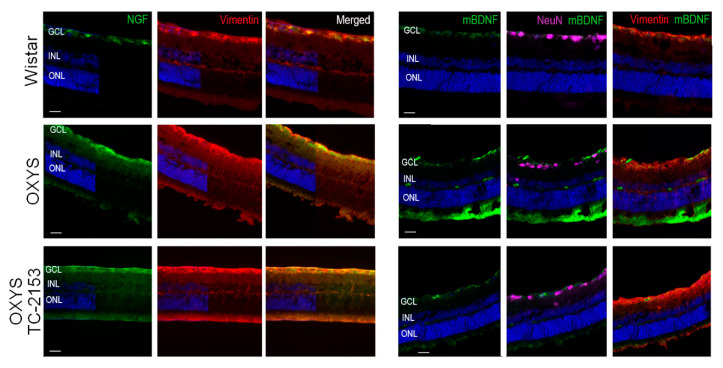
Effects of the STEP inhibitor (TC-2153) on mBDNF and NGF expression in 13-month-old OXYS rats. Representative images of a Wistar or OXYS retina stained with antibodies against NGF, mBDNF, NeuN, and vimentin. Cell nuclei were stained with DAPI. Scale bar = 20 µm. GCL, ganglion cell layer; INL, inner nuclear layer; and ONL, outer nuclear layer.

To detect a macroglial response, we utilized antibodies to GFAP and vimentin. Upregulation of GFAP is a well-established indicator of retinal injury and reactive gliosis. Vimentin is an intermediate filament that is ubiquitously expressed in Müller cells. In agreement with our previous study [11], we noted major upregulation of GFAP in OXYS rats in comparison with Wistar rats. Nevertheless, we did not reveal obvious changes in GFAP and vimentin expression in the retina of OXYS rats treated with TC-2153 in comparison with the untreated rats (Figure 2d and Figure 3).

Earlier, our group demonstrated an increase of NGF staining in Müller cells in OXYS rats at the progressive stage of retinopathy and that the mBDNF protein is located in Müller cells in OXYS rats, whereas in the Wistar retina, mBDNF immunoreactivity was detected in Müller cells and ganglion cells [18]. Here, treatment with TC-2153 did not affect the expression and localization of proteins NGF and mBDNF as compared with the untreated OXYS group (Figure 3).

## 3. Discussion

The main aim of this study was to verify the possible contribution of STEP expression alterations in the retina to the development of AMD-like pathology in OXYS rats. It is known that STEP is specifically expressed within neurons of the central nervous system (CNS), and the level of its expression significantly depends on the brain region [3]. The highest expression of STEP is seen within the striatum, while it is also expressed in other brain areas: the neocortex, amygdala, hippocampus, optic nerve, and embryonic spinal cord [4,19,20,21,22]. Two major isoforms of STEP, STEP46 and STEP61, have different patterns expression in brain regions and at developmental time points [21,23]. It should be noted that nowadays there are contradictory results on age-related STEP expression alterations in the brain [24,25], whereas information on such changes in the retina is missing.

Here, for the first time, we estimated the mRNA and protein expression of STEPs in the retina of Wistar and OXYS rats (the latter develop AMD-like retinopathy) and investigated the influence of aging on the retina. We demonstrated that there were no significant interstrain differences in the *Ptpn5* mRNA level at the age of 13 months when the clinical signs of AMD in OXYS rats were pronounced. Nonetheless, at the same time, the *Ptpn5* expression in the retina of both Wistar and OXYS rats at the age of 13 months was significantly higher than that at the age of 20 days (in the preclinical stage of AMD-like retinopathy). These results are consistent with our RNA sequencing data [16,26]. The elevated *Ptpn5* mRNA level in the retina of 13-month-old rats positively correlated with the data on its protein levels obtained in this study. At age 20 days, STEP46 and STEP61 protein levels in the OXYS retina were significantly higher than those in Wistar rats. By the age of 13 months, the STEP46 protein level increased in both strains, whereas the STEP61 protein level stabilized in Wistar rats and significantly diminished in OXYS rats. No difference was found in STEP61 levels between Wistar and OXYS rats at the age of 13 months. The age-dependent increase in STEP46 but not in STEP61 protein levels in the rat retina is of considerable interest.

Today, most studies are focused on the STEP61 isoform, and little is known about alterations of STEP46 expression with age in various tissues. Meanwhile, it has been shown that these isoforms have different patterns of expression in ontogenesis [21,23]. For instance, rodent studies have revealed that cytosolic STEP46 is first detected at P6, and its amount progressively increases until adulthood, whereas membrane-associated STEP61 begins to be expressed at birth, and its expression continues throughout adulthood in the striatum [23]. In the mouse cortex, STEP61 expression increases substantially during the first 1–3 postnatal weeks, whereas in the hippocampus, STEP61 expression peaks and stabilizes at P7 [21]. It is known that by the age of 20 days, postnatal retinal maturation is completed in rodents [27]. In addition, two active STEP isoforms (STEP61 and STEP46) have substrate specificity. For example, STEP46 interacts much more strongly with phosphorylated ERK1/2 than does STEP61, whereas STEP61 binds with higher affinity to Pyk2 and Fyn [20,28]. On the other hand, an increased STEP46 amount may be associated with a disruption of the proteasomal degradation machinery, whereas aged OXYS rats are characterized by decreased reactivity of autophagy [29,30].

In the present study, a dramatic decrease in both total phosphatase and STEP phosphatase activities was observed in the retina of 20-day-old OXYS rats. With age, total phosphatase activity and phosphatase activity of STEP increased in OXYS rats, whereas in Wistar rats, the total phosphatase activity decreased and STEP activity did not change. Overall, we detected an increase in STEP protein levels and a decrease of total phosphatase and STEP phosphatase activities in the retina of 20-day-old OXYS rats. These conflicting results may be associated with an age-related increase in the dimerization of STEP resulting in a substantial loss of phosphatase activity [31].

In OXYS rats, STEP levels increased in the retina by the age of 20 days in parallel with increased colocalization of proBDNF and p75NTR proteins [18] and an increase in apoptosis [16]. Similar alterations of BDNF expression and apoptosis have been registered in the cortex [32,33] and hippocampus [34] of OXYS rats at age 20 days. These features of OXYS rats’ phenotype may be related to the retardation of CNS development in OXYS rats at an early age. It is documented that STEP proteins are negative regulators of synaptic strengthening via dephosphorylation of regulatory tyrosine residues on their substrates [3].

As determined elsewhere, by the age of 20 days, postnatal retinal maturation is completed in rodents. In addition, two active STEP isoforms (STEP61 and STEP46) have substrate specificity. Therefore, we propose that higher STEP levels and lower STEP activity found in the retina of OXYS are likely related to the impaired neurogenesis or reduced neuronal maturation in the CNS of these animals.

According to the present results on age-related upregulation of STEP and its well-known involvement in neurodegenerative diseases, we decided to estimate the effects of prolonged STEP-specific inhibition (by TC-2153) on the OXYS rat retina. It has been previously reported that administration of TC-2153 significantly improves cognitive and behavioral function in a transgenic mouse model of AD [35,36] and in neurocognitive diseases [12,13,37]. It should be noted that in all previous published studies on rodents, the administration of TC-2153 was acute or not longer than 20 days [3,15].

Here, we administered TC-2153 for four months orally (with food). Paradoxically, no significant differences were found in STEP46 and STEP61 mRNA levels and protein expression, total phosphatase activity, and phosphatase activity of STEPs in OXYS rats. This phenomenon may be associated with a compensatory effect and/or a nonoptimal dose of TC-2153. Nevertheless, it is worth noting that STEP expression levels are also regulated by ubiquitination and degradation. Synaptic NMDA receptor activation induces rapid STEP polyubiquitination, which leads to proteasomal degradation [38]. Therefore, when synaptic activity is high, STEP levels are kept low by NMDA receptor activation and proteasomal degradation [39]. There is evidence that synaptic activity can also cause rapid synthesis of specific STEP isoforms. For example, Olausson et al. have shown that within minutes after fear conditioning, STEP46 is rapidly synthesized in neurons of the lateral amygdala (where STEP46 is not normally detectable), and increased levels of STEP46 correlate with the subsequent dephosphorylation and inactivation of ERK1/2 [40].

Our data indicate that prolonged chronic STEP inhibition in OXYS rats resulted in small but significant alterations of retinal morphometric parameters. For instance, relative thickness of the IPL was greater, whereas relative thickness of the INL was lower in TC-2153–treated OXYS rats than in untreated OXYS rats. By contrast, relative thickness of the retina and OPL did not change. The IPL contains axons of bipolar cells, dendrites of ganglion cells, and processes of amacrine cells forming synaptic contacts. STEP is a negative regulator of synaptic strengthening, and STEP inhibition leads to IPL lengthening in aged OXYS rats. On the other hand, in our study, chronic STEP inhibition caused thinning of the INL, while apoptosis magnitude did not change. We suggest that the INL thinning is associated with increasing autophagy and/or necrosis [41]. The INL consists of amacrine, bipolar, and horizontal interneurons and Müller glia bodies. We detected a significantly decreased number of parvalbumin-positive amacrine neurons in the OXYS retina after chronic TC-2153 treatment compared with both Wistar and OXYS untreated rats. Antibodies against parvalbumin predominantly stain AII amacrine cells [17]. AII amacrine cells are crucial interneurons of the light signal transduction pathway because these cells provide outputs to ON- and OFF-cone bipolar cells via glycinergic synapses. Besides, AII amacrine cells express functional NMDA receptors, which commonly mediate activity-dependent changes in postsynaptic neurons [42]. In the rat retina, NMDA and AMPA receptors are expressed also by ganglion, bipolar, and horizontal neurons as well as photoreceptors and Müller cells [43,44]. Nonetheless, we did not detect alterations in amounts of ganglion cells, photoreceptors, and Müller glia after TC-2153 treatment. OXYS rats at the progressive stage of disease are characterized by gliosis in Müller cells; for example, there is evidence of an increase in NGF staining and mBDNF localization in this type of cell in OXYS rats as compared to the Wistar strain [18,45]. Previously, it has been demonstrated that 2-week TC-2153 administration elevates the BDNF level in the brain [46]. Here, the four months of treatment with TC -2153 exerted no effect on the expression and localization of NGF and mBDNF in OXYS rats.

The decrease in the amount of parvalbumin-positive AII amacrine neurons in OXYS rats’ retina after TC-2153 treatment may be due to several factors. First, there is a possible influence of prolonged STEP inhibition on neuromediators. Research has revealed that STEP61 is a key molecule that relays signals from GABAergic neurotransmission in the spinal dorsal horn [47], and that pharmacogenetic modulation of STEP by TC-2153 decreases the serotoninergic 5-HT2A receptor protein level in the hippocampus and frontal cortex [13]. Second, parvalbumin is a protein that binds calcium with high affinity. Pharmacological inhibition of STEP may contribute to synaptic dysfunction possibly owing to perturbed synaptic Ca^2+^ handling in response to overactivation of NMDA receptors [48]. Excessive stimulation of glutamatergic signaling is excitotoxic. In turn, glutamate excitotoxicity contributes to delayed slowly evolving neurodegeneration [49]). Third, decreased AII amacrine cells numbers in OXYS rats’ retina after TC-2153 treatment may be a possible compensatory change that is not directly related to STEP. Finally, TC-2153 is considered a potential H_2_S donor capable of modulating the cell’s redox homeostasis dysfunction [50], which also contributes to AMD pathogenesis.

In summary, for the first time, we demonstrated age-dependent changes of *Ptpn5* mRNA expression, STEP46 and STEP61 protein levels, and their phosphatase activity in the retina. It was established that an increase in the STEP protein level and a decrease in total and STEP phosphatase activities in the retina precede the manifestation of clinical signs of AMD in OXYS rats. Meanwhile, there were no differences in these retinal parameters between 13-month-old OXYS rats (which manifest pronounced signs of AMD) and Wistar rats. Pharmacological inhibition of STEP with TC-2153 for four months during the active progression of AMD-like retinopathy had a negative effect on relative thickness of the retinal INL, as evidenced by a decrease in the amount of parvalbumin-positive amacrine neurons. By contrast, long-term treatment with TC-2153 had no effect on the *Ptpn5* mRNA expression, STEP46 and STEP61 protein levels, and their phosphatase activity in the retina of OXYS rats. These findings indicate that TC-2153 may exert a negative action on the retina through mechanisms not related to STEP.

## 4. Materials and Methods

### 4.1. Ethics Statement

All animal procedures were in compliance with the Association for Research in Vision and Ophthalmology statement for the Use of Animals in Ophthalmic and Vision Research and the European Communities Council Directive 86/609/EES. All manipulations of the animals were approved by Scientific Council 9 of the Institute of Cytology and Genetics, SB RAS, in accordance with the Directive 2010/63/EU of the European Parliament and the Council as of 22 September 2010. All experiments were approved by (and conducted in accordance with the guidelines of) the Ethics Committee on animal testing of the Institute of Cytology and Genetics, Novosibirsk, Russia (the decree of the Presidium of the Russian Academy of Sciences No. 12000-496 of April 2, 1980).

### 4.2. Animals and Experimental Procedures

Male senescence-accelerated OXYS rats and parental rats (control Wistar strain) were obtained from the Breeding Experimental Animal Laboratory of the Institute of Cytology and Genetics, SB RAS (Novosibirsk, Russia; RFMEFI61914X0005 and RFMEFI61914X0010). The OXYS strain was derived from the Wistar strain of rats at the Institute of Cytology and Genetics as described earlier [9]. The rats were kept under conventional conditions on a 12:12 h light/dark cycle (at 8 a.m., lights on) and had *ad libitum* access to feed pellets (PK-120-1; Laboratorsnab, Ltd., Moscow, Russia) and water unless stated otherwise.

To study the effect of age on STEP expression and phosphatase activity, we used untreated OXYS and Wistar rats at ages 20 days (*n* = 5 for each genotype) and 13 months (*n* = 10 for each genotype).

To evaluate the influence of the STEP inhibitor (TC-2153, 8-(trifluoromethyl)-1,2,3,4,5-benzopentathiepin-6-amine) on the development of AMD, OXYS rats at the age of nine months (*n* = 22) received TC-2153 orally (with food; the inhibitor was diluted with 2% Tween 20) at a dose of 10 mg/kg for six consecutive days in each week for four months until the rats reached the age of 13 months.

All rats (untreated 20-day-old and 13-month-old OXYS and Wistar rats as well as 13-month-old TC-2153–treated OXYS rats) were euthanized on the same day by CO_2_ inhalation and decapitation.

After that, eyes were carefully removed. The retina from the left eye of 13-month-old untreated Wistar (*n* = 10), untreated OXYS (*n* = 10), and TC-2153–treated OXYS rats (*n* = 10) and 20-day-old Wistar (*n* = 5) and OXYS rats (*n* = 5) was placed in a microcentrifuge tube, frozen in liquid nitrogen, stored at −70 °C, and for the molecular procedure was homogenized in 300 µL of Tris buffer (50 mM, pH 7.6) at 4 °C using a motor-driven grinder (Z359971, Sigma–Aldrich, St. Louis, MO, USA). The right eyes of rats were subjected to histochemical analyses.

### 4.3. Western Blot Analysis

For assessment of the STEP protein level, 100 µL of a retinal homogenate was added to 100 µL of homogenization buffer (300 mM NaCl, 100 mM Tris-HCl pH 8, 4 mM EDTA, 0.2% of Triton X-100) with protease inhibitors (Pierce Protease Inhibitor tablets, Thermo Fisher Scientific Inc., Waltham, MA, USA 1 mM sodium orthovanadate, 2 mM PMSF) and incubated for 1 h on ice. Then, the homogenate was centrifuged for 20 min at 2000 × *g* (4 °C), and the supernatant was transferred to a clean 1.5 mL tube and kept at −80 °C.

The concentration of total protein was evaluated by the bicinchoninic acid assay (Pierce BCA Protein Assay Kit) according to the manufacturer’s instructions using an Eppendorf spectrophotometer (BioPhotometer plus, Eppendorf, Hamburg, Germany) followed by adjustment of the concentrations to the same level with 2 × Laemmli sample buffer (4% of sodium dodecyl sulfate (SDS), 20% of glycerol, 120 mM Tris-HCl pH 6.8, 10% of β-mercaptoethanol, 0.02% of bromophenol blue). The protein samples were denatured by boiling for 5 min at 95 °C. The proteins (30 µg per lane) were resolved by electrophoresis on an SDS 10% polyacrylamide gel and were blotted onto a nitrocellulose membrane (Thermo Fisher Scientific Inc., Waltham, MA, USA). The membranes were blocked for 3 h in TBST buffer containing 5% of nonfat dry milk, then rinsed, and incubated with a polyclonal mouse anti-STEP antibody (dilution 1:1000, sc-, Santa Cruz Biotechnology, Dallas, TX, USA) at 4 °C overnight and then with a secondary goat anti-mouse IgG antibody conjugated with horseradish peroxidase (dilution 1:, sc-2031, Santa Cruz Biotechnology, Dallas, TX, USA) for 2 h at room temperature (RT). The blots were treated with the SuperSignal West Femto Maximum Sensitivity Substrate (Thermo Fisher Scientific Inc., Waltham, MA, USA) according to the manufacturer’s instructions. Protein bands were detected on a Fusion FX7-820 system (Vilber Lourmat, Marne-la-Vallée, France). Quantification of the protein bands was performed by means of volume densitometry in ImageJ software (National Institutes of Health, Bethesda, MD, USA). For reference protein staining, we chose an anti-GAPDH antibody (dilution 1:500 in 5% milk, sc-25778, Santa Cruz Biotechnology Dallas, TX, USA) for 2 h at RT. STEP61 and STEP46 protein levels were evaluated as the percentage ratio of fluorescence intensity of STEP relative to the fluorescence intensity of a GAPDH band. STEP61 and STEP46 were detected at 61 and 46 kDa positions on the blots, respectively. GAPDH was detected at 37 kDa.

### 4.4. Quantitative Reverse-Transcription PCR (qPCR)

Total RNA samples were extracted from 60 µL of the retinal homogenate using 300 µL of TRIzol Reagent (Ambion, Waltham, MA, USA) according to the manufacturer’s instructions. The extracted RNA samples were treated with RNA-free DNase (Promega, Madison, WI, USA) and diluted to 0.125 µg/µL with diethyl pyrocarbonate–treated water. The obtained total RNA was subjected to cDNA synthesis with a random hexanucleotide mixture (BioLab Mix, Novosibirsk, Russia). To evaluate the number of *Ptpn5* cDNA copies, we performed SYBR Green I fluorescence detection (R-402 Master mix, Syntol, Novosibirsk,, Russia) with primers specific to STEP46 and STEP61 isoforms (forward primer: 5′-CGTGGTAGACATCCTAAAGACC-3′, reverse primer: 5′-CTGATACTGTTCGCATGTTTGG-3′) with respect to 100 copies of a housekeeping gene: DNA-dependent RNA polymerase II (*Polr2a*) (forward primer: 5′-TTGTCGGGCAGCAGAACGTG-3′, reverse primer: 5′-CAATGAGACCTTCTCGTCCTCCC-3′). As external standards, we used genomic DNA (0.5, 1, 2, 4, 8, 16, 32, and 64 ng/µL) extracted from the liver of male Wistar rats [51,52]. To monitor amplification specificity, we performed melting curve analysis at the end of each run for each primer pair.

### 4.5. A Real-Time Spectrophotometric Assay of Phosphatase and STEP Activities

The retinal homogenate (140 µl) was centrifuged (#5430R, Eppendorf, Hamburg, Germany) for 20 min at 12,700 rpm (4 °C). Phosphatase and STEP activities were measured in the cleared supernatant by a recently developed spectrophotometric method [53]. The total concentration of protein in the supernatant was measured by the Bradford assay (#500-0006, Bio-Rad, Hercules, CA, USA) according to the manufacturer’s instructions. The incubation mixture consisted of 100 µL of 2-(N-morpholino) ethanesulfonic acid (MES; Serva, Heidelberg, Germany) buffer (50 mM, pH 7.0), 20 µL of a supernatant (1.5 µg/mL of total protein diluted with MES buffer), and 30 µL of a p-nitrophenylphosphate solution (8 mM p-nitrophenyl phosphate disodium salt hexahydrate (Acros Organics, Fair Lawn, NJ, USA) diluted with MES buffer). The reaction was carried out in a Costar assay plate (#9018, Corning, New York, NY, USA) on a Multiscan GO spectrophotometer (Thermo Fisher Scientific Inc., Waltham, MA, USA). As a standard for the calibration curve, we employed 20, 10, 5, and 2.5 µM 4-nitrophenol (Merck, Kenilworth, NJ, USA) diluted with MES buffer (50 mM, pH 7.0). The samples were incubated for 40 min at 35 °C. This procedure assays the total activity of all phosphatases in a sample (including STEP) (V_1_ in the formula below). After 40 min of the incubation, 5 µL of TC-2153 diluted as described earlier [13,46] was added to the wells to a final TC-2153 concentration of 10 µM, and the microplate was incubated for another 40 min at 35 °C. This procedure assesses the activity of phosphatases without STEP in the sample (V_2_ in the formula below). Optical densities of the samples were registered at 405 nm every 5 min during both incubations. The activity of all phosphatases was expressed in nanomoles of products per milligram of total protein (P) per minute (nmol/[mg⋅min]). The STEP activity, nmole/(mg⋅min), was calculated as follows:

V_STEP_ = (V_1_ − V_2_)/P (1)

Only freshly prepared p-nitrophenyl phosphate, 4-nitrophenol, and TC-2153 were used in the total phosphatase and STEP assays.

### 4.6. Immunohistochemistry and the TUNEL Assay

After decapitation, right eyes were carefully removed and immersed in a freshly prepared fixative solution (4% paraformaldehyde diluted in 0.1 M phosphate buffer [PB, pH 7.4]) for 2 h at RT. After extensive rinsing in 0.1 M PB, a cryoprotection solution (10%, 20%, and 30% sucrose diluted in 0.1 M PB) was applied overnight at 4 °C, and then the eyecups were embedded in a tissue-embedding medium (Shandon Cryomatrix, Thermo Fisher Scientific Inc., Waltham, MA, USA). Next, 10-μm-thick cryosections were cut off vertically and stored at −20 °C until analysis. Immunohistochemical procedures were performed on the cryosections according to protocols previously published by our laboratory [11]. In brief, prior to incubation with primary antibodies, to block nonspecific staining, the sections were treated with 5% bovine serum albumin diluted in phosphate-buffered saline (PBS, pH 7.4) supplemented with 0.1% Triton X-100 for 2 h at RT. Primary antibodies (anti-NeuN, cat. # ab177487; anti-Parvalbumin, ab11427; anti-GFAP, ab7260; anti-vimentin, ab24525; and anti-NGF, ab6199; all at 1:100 dilution; Abcam, Cambridge, United Kingdom; and anti-mBDNF, GF35L-100 UG, 1:250, Millipore, Burlington, MA, USA) were added to probe the sections overnight at 4 °C. After repeated rinsing, species-specific fluorescent probes (Alexa 488, Alexa 594, and Alexa 647 conjugates, 1:200, Abcam, Cambridge, United Kingdom) were applied for 1 h at RT. Cell nuclei were counterstained with DAPI (Abcam, ab104139, Cambridge, United Kingdom). Sections with the primary antibodies omitted served as negative controls.

Neural apoptosis was assessed by TUNEL staining with a DeadEnd Fluorometric TUNEL System (cat. # G3250, Promega, Madison, WI, USA). A standard TUNEL procedure was performed as previously described [16]. The cell nuclei were stained with DAPI.

### 4.7. Measurement of Retinal Thickness and Cell Counting

The counting was performed in a blinded experiment; the names of retinal-section groups were not known to the observer until after the counting procedures were completed.

These procedures were performed on 10-μm-thick vertical cryosections (three sections per specimen) obtained from TC-2153–treated OXYS (*n* = 4) and untreated Wistar (*n* = 4) and OXYS (*n* = 4) rats. The cryosections were analyzed under an Axioplan 2 microscope (Carl Zeiss SMT GmbH, Oberkochen, Germany) with a 20 × objective.

Only the sections going through/near the optic nerve head were selected.

To evaluate retinal thickness, we analyzed long central (immediately adjacent to the optic nerve head or central) and mid-peripheral (approximately three fields away from the optic disc or central) regions in both superior and inferior directions.

To assess the impact of treatment on retinal thickness, we analyzed distances between the ganglion and outer nuclear layers (GCL–ONL), IPL, INL, OPL, and ONL in six sections per retina derived from four specimens from each group. To standardize all retinal layers’ thickness values, the data were normalized to Wistar rats’ retinal thickness; the latter was set to 100%. In the ONL, we counted nuclei in at least three columns per location, in parallel with the thickness measurements.

Using the Zen LE software (Carl Zeiss SMT GmbH, Oberkochen, Germany), total numbers of stained cells per section were determined and averaged as described in ref. [17]. In brief, in the enumeration of AII amacrine cells (they usually stain with the anti-parvalbumin antibody) and ganglion cells (they typically stain with the anti-NeuN antibody), the total number of the stained cells throughout the whole length of a retina per section was determined. Parvalbumin-positive cells were counted in the INL, and NeuN-positive cells were counted in the GCL. For quantitative analysis of certain markers (GFAP, NGF, and BDNF, through immunostaining), retinas without peripheral regions were chosen in both superior and inferior directions. At least four tissue slices (biological replicates) were analyzed per animal. Among all image acquisition procedures, all imaging parameters were the same.

### 4.8. Statistics

The effects of age and genotype on the phosphatase activities and mRNA and protein levels were analyzed by two-way ANOVA (factors: age and genotype). The differences between groups were assessed via Fischer’s LSD *post hoc* multiple pairwise comparisons. The effects of chronic TC-2153 treatment on all the studied parameters were subjected to one-way analysis of variance (ANOVA in Statistica 8.0 software, Palo Alto, CA, USA). The data are presented as the mean ± SEM. Data with P values less than 0.05 were considered significant.

## 5. Conclusions

In summary, we first showed age-dependent alteration of STEP46 and STEP61 expression in the retina of rats. We noted age-dependent upregulation of the STEP46 protein but not the STEP61 protein in the retina of Wistar and OXYS rats. The imbalance between levels and activities of STEP46 and STEP61 at the preclinical stage (20 days) indicates a likely contribution of these tyrosine phosphatases to the development AMD-like retinopathy in OXYS rats. We found that the prolonged chronic pharmacological inhibition of STEPs by TC-2153 in OXYS rats caused a decrease in the thickness and in parvalbumin-positive-neuron content of the INL. Our results point to functional significance of STEP expression for retinal neurons and an association of STEP with AMD pathogenesis. Further studies on the role of STEP in aging and progression of neurodegenerative diseases are needed.

## Figures and Tables

**Table 1:** The impact of chronic TC-2153 administration on total and STEP phosphatase activities as well as on STEP protein and *Ptpn5* mRNA levels in the retina. Data are presented as the mean ± SEM.

	Wistar	OXYS	OXYS TC-2153	F, p
Total phosphatase activity, nmole/(mg·min)	0.997 ± 0.039	0.986 ± 0.031	1.044 ± 0.038	F_2,21_ = 0.74
STEP phosphatase activity, nmole/(mg·min)	0.729 ± 0.036	0.720 ± 0.031	0.773 ± 0.028	F_2,21_ = 0.80
*Ptpn5* mRNA level, fold change relative to Wistar rats	1.00 ± 0.070	1.052 ± 0.119	1.062 ± 0.083	F_2,21_ = 0.13
STEP46 protein level, a.u.	92.24 ± 17.98	92.13 ± 14.32	118.18 ± 20.54	F_2,15_ = 0.71
STEP61 protein level, a.u.	89.04 ± 10.41	97.26 ± 12.52	101.38 ± 10.23	F_2,23_ = 0.33

## Supplementary Materials

Supplementary materials can be found at https://www.mdpi.com/1422-0067/21/15/5182/s1. Figure S1: Western blot analysis effects of the STEP inhibitor (TC-2153) on STEP proten level in retina.

## Abbreviations

AD	Alzheimer’s disease
AMD	Age-related macular degeneration
ANOVA	Analysis of variance
GCL	Ganglion cell layer
INL	Inner nuclear layer
IPL	Inner plexiform layer
ONL	Outer nuclear layer
OPL	Outer plexiform layer
P	Postnatal day
RPE	Retinal pigment epithelium
SDS	Sodium dodecyl sulfate
STEP	Striatal-enriched tyrosine protein phosphatase
TC-2153	8-(trifluoromethyl)-1,2,3,4,5-benzopentathiepin-6-amine
