# Supplementary material for: Alterations of STEP46 and STEP61 Expression in the Rat Retina with Age and AMD-Like Retinopathy Development
Source: Int J Mol Sci. 2020 Jul 22;21(15):5182. doi: 10.3390/ijms21155182 (PMC7432912; doi:10.3390/ijms21155182)

**Supplementary Figure S1.** Western blot analysis effects of the STEP inhibitor (TC-2153) on STEP protein level in retina.

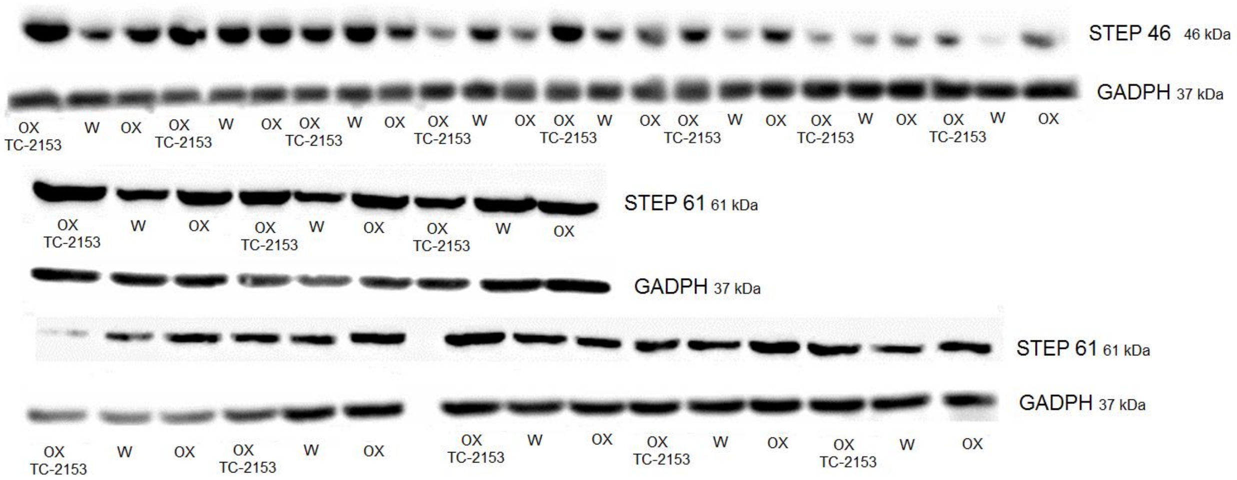

Supplement: Supplementary file 1 [file ijms-21-05182-s001.pdf]
